# A Sprayed Graphene Pattern-Based Flexible Strain Sensor with High Sensitivity and Fast Response

**DOI:** 10.3390/s19051077

**Published:** 2019-03-03

**Authors:** Wei Xu, Tingting Yang, Feng Qin, Dongdong Gong, Yijia Du, Gang Dai

**Affiliations:** 1 Institute of Electronic Engineering, China Academy of Engineering Physics, Mianyang 621900, China; xuwei@mtrc.ac.cn (W.X.); qinfengg3@mtrc.ac.cn (F.Q.); gongdongdong@mtrc.ac.cn (D.G.); duyijia@mtrc.ac.cn (Y.D.); 2 Microsystem and Terahertz Research Center, China Academy of Engineering Physics, Chengdu 610200, China

**Keywords:** graphene, flexible strain sensor, high sensitivity, fast response, curved substrate

## Abstract

Flexible strain sensors have a wide range of applications in biomedical science, aerospace industry, portable devices, precise manufacturing, etc. However, the manufacturing processes of most flexible strain sensors previously reported have usually required high manufacturing costs and harsh experimental conditions. Besides, research interests are often focused on improving a single attribute parameter while ignoring others. This work aims to propose a simple method of manufacturing flexible graphene-based strain sensors with high sensitivity and fast response. Firstly, oxygen plasma treats the substrate to improve the interfacial interaction between graphene and the substrate, thereby improving device performance. The graphene solution is then sprayed using a soft PET mask to define a pattern for making the sensitive layer. This flexible strain sensor exhibits high sensitivity (gauge factor ~100 at 1% strain), fast response (response time: 400–700 μs), good stability (1000 cycles), and low overshoot (<5%) as well. Those processes used are compatible with a variety of complexly curved substrates and is expected to broaden the application of flexible strain sensors.

## 1. Introduction

Traditional strain sensors are mainly based on metal and semiconductor materials [1,2,3], using the “strain resistance” effect to convert the local strain of the measured object into a measurable change in resistance [4,5,6]. In recent years, with the miniaturization and distributed development of sensors, traditional sensing materials have been unable to meet the comprehensive requirements of high sensitivity coefficient, fast response, flexibility, and space adaptability. Compared with traditional strain sensors, flexible strain sensors have capabilities of portability, flexibility, transparency, continuous detection and good biocompatibility [7,8,9]. At present, sensitive materials commonly used in flexible strain sensors include metal nanoparticles, nanowires, carbon nanotubes, polymer nanofibers, graphene, etc. The introduction of nanomaterials and nanostructures provides a new mechanical design idea for the construction of flexible devices, while endowing the device with rich functionality, which can be widely used in human health assessment, flexible touch screen, an electronic skin, aerospace, as well as mechanical engineering [10,11,12,13].

Graphene has unique structural characteristics, defect dependent electrical properties and multi-dimensional spatial adaptability. It has become one of the preferred materials for achieving highly-sensitive sensing and is expected to be widely used in flexible sensor devices to detect weak mechanical stimulation [14,15]. Moreover, the adjacent graphene sheets are easy to achieve rapid non-destructive relative sliding due to its ultra-lubricating property, which is beneficial to improve the response speed of devices [16]. Through the design of graphene microstructures and the pattern topology structure of graphene, not only the performance of the sensor can be improved [17,18,19,20], but also some specific functions can be realized [21,22,23].

As a key indicator of strain sensor performance, sensitivity is often reported in the literature [24,25,26]; however, there are few reports on response time, or just as a supplement to other performance. Notably, the response time here refers to that of the whole flexible strain sensor, mainly including the time when stress wave passes through the substrate, the time when strain is coupled to the sensitive layer, and the time when the sensitive material transforms the deformation into a simple output signal. According to the available literature reported in the past five years, the response time of flexible strain sensors is still in the order of milliseconds [27,28,29,30,31], which is obviously unsuitable for those applications where fast response speed is demanded. In addition, the fabrication of some flexible strain sensors previously reported requires deposition, evaporation, ion etching, etc. Those processes are complex and costly. Therefore, this paper uses graphene solution as raw material, realizes patterning of graphene by simple oxygen plasma treatment and spraying process combined with a soft mask, and obtains flexible strain sensor with good space adaptability. Through further parameter optimization, high sensitivity and fast response of the sensor devices are simultaneously achieved. Furthermore, the working mechanism of the sensor from the micro-level has been explored. Compared with the previous work, we give a more effective and convenient method to tune the sensitivity of the sensor by the design of the interfacial bonding strength as well as the pattern topology structure of the graphene film. Results show that under the same condition (initial resistance: ~11 kΩ, strain: 1%), the sensitivity of the sensor can be increased by over six times than that of the previous one [2].

## 2. Materials and Methods

### 2.1. Substrate Preparation

Here we chose a common flexible substrate material–polydimethylsiloxane (PDMS) as an example. The base agents (SYLGARDTM 184 silicone elastomer base) of PDMS were mixed with curing agents (SYLGARDTM 184 silicone elastomer curing agent) at the ratio of 10:1. The mixture was stirred with a blender for 2 min immediately and then treated in vacuum for 5 min to eliminate bubbles in the mixture. Four grams of the mixture was taken in a Petri dish (125 mm of diameter) and the dish was laid flat for over 24 h at room temperature to cure the PDMS.

### 2.2. Sensor Preparation

**Figure 1:**
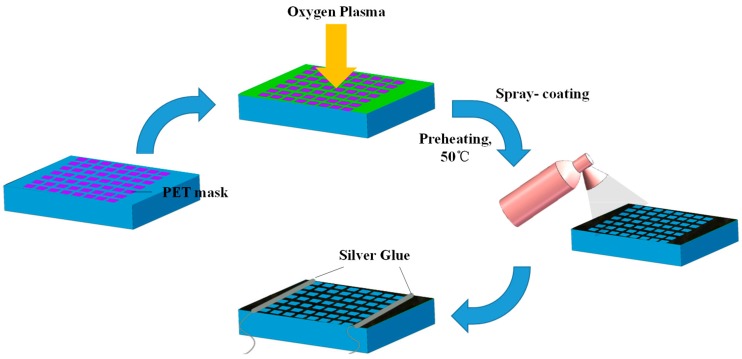
Schematic illustration of graphene flexible strain sensor fabrication process.

The whole fabrication process of graphene flexible strain sensor based on PDMS substrate is shown in Figure 1. The PDMS film was cut into the size of 20 mm × 10 mm and covered with a soft poly ethylene terephthalate (PET) matrix mask, which had predefined patterns. In order to arrange the PET rectangular mask matrix more effectively and neatly, under the Petri dishes with PDMS substrates, coordinate papers were laid flat, and the PET masks were arranged on the substrate in file according to the coordinates on the paper. The substrate and mask were put together into the oxygen plasma cleaner for 5 min. Besides, the treatment time was a key parameter affecting the performance of sensor samples. A certain degree of bombardment can add more oxygen-containing functional groups to the surface of the substrate, while it also destroys the surface structure of the base after long-term treatment, seriously affecting graphene assembly on the surface subsequently. Oxygen plasma treatment aims at introducing oxygen-containing groups, causing a strong chemical bonding reaction between the substrate and graphene and enhancing the interfacial interaction as a result.

The graphene sensing layer was fabricated by spray-coating. The graphene dispersion (solvent: 1-methylpyrrolidone (NMP), XFNANO #102084) is commercially available with a density of 2 mg/mL and a sheet diameter of over 200 nm. Before deposition, we preheated the substrates at 50 °C and then took about 0.1 mL of the graphene dispersion into the spray gun, and fixed the gun at a distance of about 150 mm away from the sample center and ensured the gun was perpendicular (90 °C) to the sample. Then, we sprayed the graphene dispersion at a constant speed of 0.01 mL/s for 10 s. After drying for 24 h at the room temperature, we removed the PET mask and obtained the graphene grid film finally. The number of graphene layers in the flake was about 1–7. The thickness of the deposited graphene film was measured by a probe profiler (Dektak XTL, Bruker) showing that the thickness of the graphene sensing layer after spray coating was about 355 nm, shown in Figure S1. Spray-coating is a commonly used coating method, which can achieve low cost, large area fabrication, and can even be applied to the manufacture of sensitive layers on complex non-planar surfaces. The properties of these graphene films can be adjusted simply and conveniently by changing the process parameters. For example, by changing the spraying-coating parameters or the oxygen plasma treatment time, we can get corresponding graphene sensing layers with adjusted performance. Silver glue was coated on both sides of the graphene film to connect silver wires which function as the electrical interconnection. At last, we achieved the soft strain sensor with a size of 20 mm × 10 mm, of which the effective working area of the sensing layer was 10 mm × 10 mm.

## 3. Results and Discussion

### 3.1. Structure of Graphene Flexible Strain Sensor

**Figure 2:**
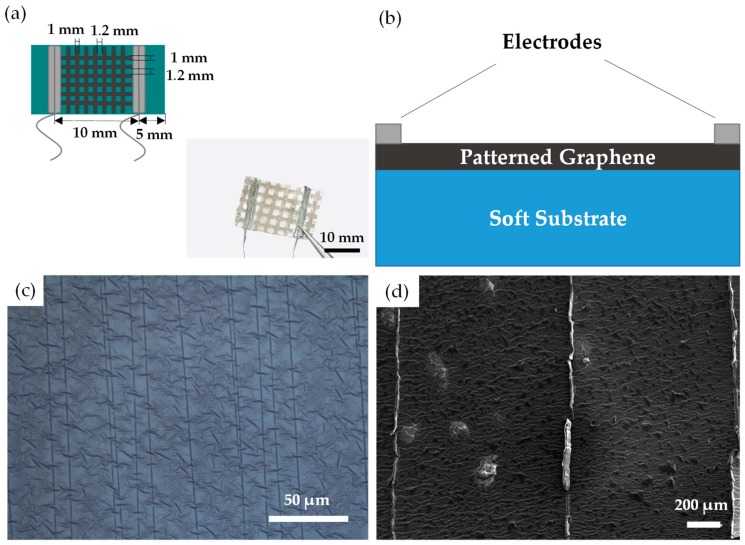
(**a**–**b**) Photograph and schematic images of the strain sensor using polydimethylsiloxane (PDMS) substrate; (**c**) optical image of PDMS substrate treated by oxygen plasma after 10 pre-stretch cycles (0–10% strain range); (**d**) SEM image of the graphene film on PDMS after 500 loading and unloading (0–1% strain range) cycles.

As Figure 2a,b shows, the flexible strain sensor mainly consists of three parts: a soft substrate, sensitive layer, and electrodes. As mentioned in Section 2.2 above, the overall sensor size was 20 mm × 10 mm. The electrodes are connected on each side of the sensor, and the effective sensitive layer area was about 10 mm × 10 mm. In Figure 2a, all the widths of the graphene strips were 1 mm and the gap between strips was 1.2 mm. According to Figure 2b, the strain or stress wave was applied to the flexible substrate and caused corresponding substrate deformation at first. Then it was coupled to the sensitive layer through the interface, and finally converted into an electrical signal to get transport. That is to say, the interfacial effect, especially the interface bonding between the substrate and the sensitive layer plays a key factor affecting the sensing performance of the sensors. Enhanced interfacial interaction helps to further optimize the response time, sensitivity, stability, and other performance of the devices. Therefore, oxygen plasma treatment was applied to modify the surface of the substrate to enhance the bonding between the substrate and the graphene layer. 

In order to better illustrate the relationship between pretreatment parameters and sensor performance, we designed a series of experiments to visually characterize the graphene layer morphology under different oxygen plasma pretreatment time, which are displayed in Figure S2. From Figure S2, the sensor without oxygen plasma treatment cannot form a continuous graphene film on the substrate. After 5 min of oxygen plasma treatment, the substrate surface had good interface energy, and a complete uniform and flat graphene sensitive layer film formed on the surface. The substrate treated by plasma for 30 min leads to the formation of vertical cracks extending in depth and even brittleness of the substrate material. The deposited graphene film was not uniform and continuous.

On the other hand, oxygen plasma treatment changes the surface mechanical property of the substrate, and directional cracks will occur under substrate tension of 10% strain as shown in Figure 2c. The graphene film had no cracks when deposited on the substrate at the beginning, but after 500 loading and unloading (0–1% strain range) cycles, a series of directional cracks were produced perpendicular to the direction of stress, as shown in Figure 2d. Because the surface energy of the substrate was obviously increased after modification of oxygen plasma, and the interaction between the substrate and the sensitive layer was greatly enhanced as well. When tensile strain was exerted to the whole sensor, directional cracks will form on the substrate correspondingly, which caused local stress concentration around the cracks. Since the interface bonds well, the stress on the substrate will be effectively coupled to the sensitive layer. The more concentrated stress will initiate cracks in the corresponding position of the sensing layer, and eventually form the similar crack morphology on the sensing layer as that on the substrate. The directional cracks in graphene film indicate the strain of the substrate has been effectively transferred to the graphene layer due to good interfacial interaction. Without modification, interfacial bonding mainly depends on intermolecular forces. In this case, the deformation cannot be effectively transmitted to the sensitive layer, and the response time is relatively long. With the increase of modification time, the interfacial bonding strength augments. Most of the deformation is transferred to the sensing layer, mainly released by the slip between graphene flakes, which quickly triggers the resistance change of the sensing layer and eventually achieves high sensitivity and fast response time. However, excessive interface modification makes the sensitive layer non-uniform and non-continuous, which is not conducive to the practical application of graphene strain sensors. Therefore, moderate modification (oxygen plasma for 5 min in this work) of the interface can not only protect the device’s structure but also obtain good performance, like high sensitivity, fast response time, etc.

### 3.2. Test of Flexible Strain Sensor with High Sensitivity and Fast Response

The overall performance of the sensor is affected by the topological structure of the macro-patterned graphene film and the micro stacking structure between the graphene sheets [27,32,33,34]. However, in order to improve a specific performance index, another performance index might be compromised. Besides, there are few studies on the response time of flexible strain sensor in the literature. In fact, the response time of the previously reported flexible strain sensor is usually on the order of milliseconds, which is far inferior to that of the traditional strain sensor. 

**Figure 3:**
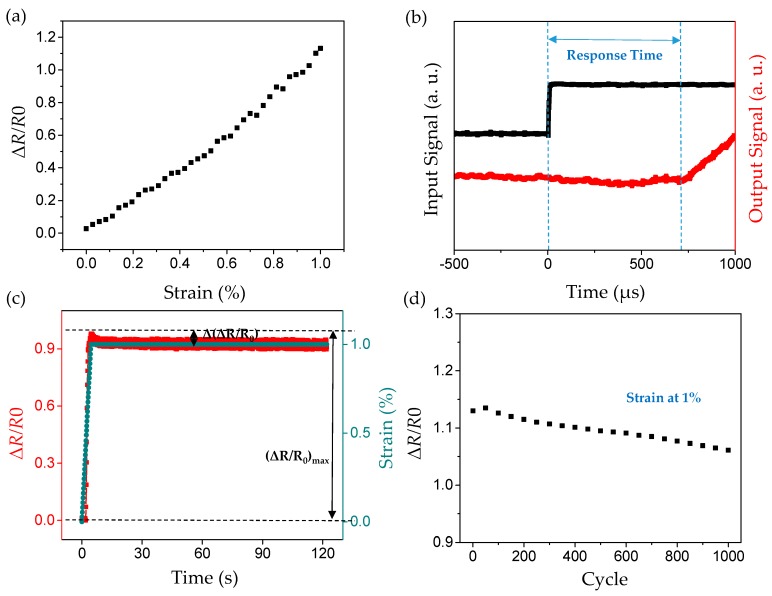
The flexible strain sensor shows high performances. (**a**) Resistance changes of the strain sensor under 0–1% strain at the 1000th cycle of loading/unloading test; (**b**) the response time is within the range of 400 to 700 μs under the impact acceleration of 500 g; (**c**) the measurement of the creep characteristic, blue curve represents the strain measurement curve of sensor with time and the red one is the actual ΔR/R0 measurement curve; (**d**) ΔR/R0 value of the sensor at strain 1% under 1000 cyclic measurement of 0–1% strain, sampling every 50 cycles.

Interestingly, following the fabrication process mentioned above, we can get strain sensors with a gauge factor of over 100 in the 0–1% strain range and a response time within the range of 400 to 700 μs. The sensor was clamped on the mechanical property testing platform (INSTRON 3380) and slowly stretched at a fixed rate (generally 0.05%/s in this experiment). The change of strain with time was recorded meanwhile the change of the resistance with time was tested by Keithley 2400. The strain–resistance curve of the sensor can be obtained by combining the strain-time curve with the resistance–time curve, as Figure 3a shows. Gauge factor (GF), defined as the change of relative resistance under unit strain, characterizes the sensitivity of the strain sensor. The calculation formula is as follows:

(1) $$\mathrm{GF}=\Delta R/\left(R_{0}\times \varepsilon \right)$$

Thus, the corresponding gauge factors (GFs) are calculated to be >100 within 1% strain range.

The test equipment of response time is exhibited in Figure S3, which consists of three parts: input circuit, response output circuit of strain sensor and oscilloscope. The input circuit includes an impactor (weight: 50 g, insulation rigid material), a base (insulation rigid material), a dry battery (1.5 V), and a resistor (75 kΩ). During the test, the impactor was released at the distance of 15 cm from the center of the base. When the impactor hit the base, the input circuit closed, and the voltage of resistor jumped from 0 V to 1.5 V. This step signal was captured by channel 1 of the oscilloscope as the input signal. To test the response time, the sensor sample was attached to the surface of an impactor, and the deformation occurred almost instantaneously when the impactor hit the base. At the same time, the deformation causes the change of the sensor resistance, after the switch of the conversion circuit, was recorded by channel 2 as output signal. The time difference between the time when the input signal steps and the time when the resistance of the strain sensor begins to change was taken as the overall response time of the sensor. Figure 3b shows that the response time of our sensor is in the range of 400 to 700 μs and the rising time, given by Figure S4, is about 400 to 650 μs.

Creep is a phenomenon in which the resistance of the sensor changes with time while the strain is kept constant. Figure 3c illustrates that when the strain is constant (1%), the sensor resistance gradually decays with time and eventually tends to be stable, resulting in a certain degree of overshoot. The degree of overshoot characterized by the ratio of Δ(ΔR/R0) and ${\left(\Delta R/R0\right)}_{max}$ is about 4.54% as shown in the figure.

Repeatability is used to evaluate the consistency of the results obtained from repeated measurements of the same sample in a short time under the same measurement conditions. In this work, 1000 cycles of cyclic loading and unloading (0–1% strain range) of a single sample were observed and the changing trend of the test results was recorded in Figure 3d. From the figure, we can see the strain sensor has a high stability, in which the gradual attenuation of resistance change (about 7% drop after 1000 cycles) was observed during the 1000 cycles of loading/unloading.

### 3.3. Theoretical Analysis and Modeling of Strain Sensing Mechanism

**Figure 4:**
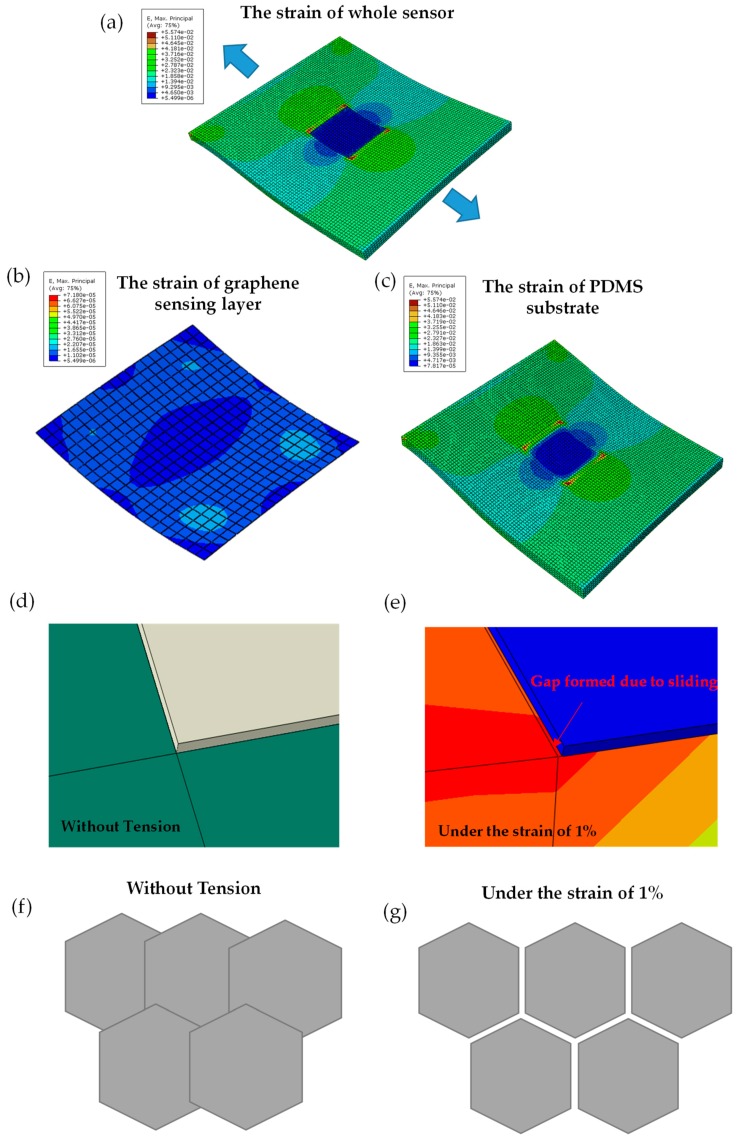
The finite element method (FEM) simulation analysis of the slip of graphene sheets when the substrate is under uniaxial stretching. (**a**) The strain distribution of the sensor (**b**) and (**c**) is the strain simulation of graphene sheet and PDMS substrate respectively; (**d**) the relative movement of the graphene sheet with substrate before stretching and (**e**) after stretching; (**f**) the schematic microscopic stacking of graphene sheets before stretching and (**g**) after stretching.

In order to understand the sensing mechanism of the sensor in the process of deformation, strain analysis of the sensor in uniaxial stretching condition was carried out, and a finite element method (FEM) mechanical model was constructed. Figure 4a shows the strain distribution of the whole sensor under uniaxial stretching. It can be seen that the graphene sheet shows nearly zero strain, while most of the strain is distributed on the surface of the non-graphene covered area of the substrate. To further illustration, the strain distribution simulation of the graphene sheet and PDMS substrate was carried out respectively, as shown in Figure 4b,c. 

As mentioned in Section 3.1, the structural characteristics of graphene-sensitive layer are as follows: in macro-scope, the graphene film is a two-dimensional patterned structure, while microscopically, graphene demonstrates the polycrystalline sheets stacking structure. Adjacent graphene sheets are connected or stacked to form a continuous film, in which the stacking width is generally 10–100 nm [35]. The elastic modulus of the graphene sheet is 5–7 orders higher than that of PDMS substrate, and the sliding friction resistance of adjacent graphene sheets is very low [36,37]. Therefore, as illustrated in Figure 4d,e, the graphene sheet will move along with the substrate during stretching and the lattice deformation of the graphene sheet can be neglected. The stretch deformation loaded on graphene mainly relies on the sliding between adjacent sheets to release. Figure 4f,g illustrate that tensile deformation will reduce the overlap area between adjacent sheets of graphene and eventually lead to complete detachment and even micro-crack. In addition, the resistivity of graphene sheet itself is eight orders of magnitude lower than that between sheets [38,39]. Therefore, the total resistance of graphene film is mainly determined by the resistance between graphene sheets [20].

The enhanced interface bonding caused by oxygen plasma pretreatment may propose fast response of sensors. We designed experiments to visualize the interface bonding strength under different pretreatment parameters. We have obtained three kinds of sensors with different oxygen plasma treatment time. The specific operation steps are exactly the same as those mentioned in the previous paragraph. Then, adhesion test tape (# 3M 600, Scotch) was slowly and smoothly covered on the surface of the sensing graphene layer of all three sensors, and 0.5 N force was applied evenly on the effective area of the tape for 5 s. Used the stretcher (INSTRON 3380) to clamp one end of the tape and tore the tape at a uniform speed of 0.08 mm/s. The test results are shown in Figure S5. The interface bonding strength of the substrate without plasma treatment was the worst, and the graphene layer on the substrate was completely torn off by the tested tape. After 5 min of oxygen plasma treatment, the interfacial bonding strength was moderate, most of the graphene layers on the substrate were torn off, and some graphene layers remained on the substrate. After 30 min treatment, the bonding strength of the interface was strong, and only a few graphene layers on the substrate were torn off. These experimental results show that oxygen plasma treatment was indeed beneficial to enhance the interface bonding, which further promotes the sensors to produce fast response.

On the other hand, as mentioned in Section 3.1, the graphene film inherited the fracture behavior of the PDMS substrate, demonstrating penetrating cracks in a direction perpendicular to the direction of stretch, which prompted the rapid change of graphene resistance and produced a more sensitive and faster response. In summary, it is attributed to the directional cracks of the sensing layer in macro, the slip of graphene sheets in micro, as well as the enhanced interface bonding, which together bring about high sensitivity and fast response of the graphene strain sensor.

### 3.4. Application of the Flexible Strain Sensor 

**Figure 5:**
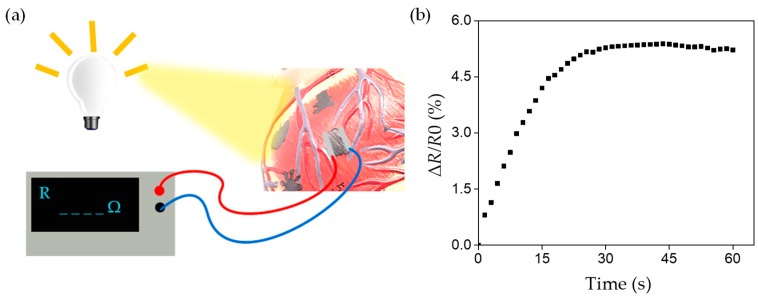
The sensors are applicable to detect complex curved surfaces. (**a**) Illustration of micro-strain detection on the complex model surface under light illumination; (**b**) The change of ΔR/R0 of sensor with illumination time.

Our flexible strain sensor not only has high sensitivity, fast response, and other excellent performance, but also can conformally cover non-planar objects to effectively achieve the detection task on complex surfaces. To better illustrate, we give a practical case to exhibit that sensors fabricated by this process are fully competent for detecting complex curved objects. As shown in Figure 5a, the graphene sensitive layer was deposited directly on the partial surface area of a standard heart model. This structure is challenging for accurate monitoring, because of irregularity, complexity and low deformability on its surface. A light source was used to illuminate the surface of the model for 60 s continuously and uniformly. The whole process of the sensor resistance changing with the illumination time was recorded by digital source meter. Due to the illumination, the surface of the model will produce micro-deformation, which could be detected by our sensors and converted into electrical signal output. The result in Figure 5b represents that such micro strain can cause about 5.37% resistance change of the sensor, which means sensors made by the method above are completely applicable to detect such a complex curved surface. The successful implementation of the above case indicates that this sensor has the ability to detect irregular curved objects.

## 4. Conclusions

In a word, we have proposed a low-cost, convenient, common method for fabricating patterned graphene-based flexible strain sensor with high sensitivity and fast response. Through modification of partial areas on the substrate directly, the graphene pattern could be deposited quickly, uniformly and extensively, without requirements of evaporation, photolithography, transfer, etc. Meanwhile, sensors obtained by this process also show high performances. The sensors’ GF was over 100 within a strain range of 0–1% and sensors can respond within the range of 400 to 700 μs. The sensors also showed good stability and low creep. To further explain sensing mechanism, we have established the FEM mechanical model to analyze the sensor deformation behavior, which refers that graphene sheets release strain by slip instead of lattice deformation themselves. Besides, an example of practical application has been presented, which indicates this sensor has the ability of conformal coverage on non-planar objects and is suitable for accurate detection of irregular surfaces as well.

## Supplementary Materials

The following are available online at https://www.mdpi.com/1424-8220/19/5/1077/s1, Figure S1: The thickness of sprayed graphene film tested by a probe profiler, Figure S2: The surface morphology of graphene layer under different oxygen plasma treatment time, Figure S3: Schematic diagram of self-designed experimental equipment for response time test, Figure S4: The whole progress of graphene strain sensor to output the response signal, Figure S5: Interfacial bonding strength test under different oxygen plasma treatment time.
