# Supplementary material for: A Sprayed Graphene Pattern-Based Flexible Strain Sensor with High Sensitivity and Fast Response
Source: Sensors (Basel). 2019 Mar 3;19(5):1077. doi: 10.3390/s19051077 (PMC6427754; doi:10.3390/s19051077)
Supplement: Supplementary file 1 [file sensors-19-01077-s001.pdf]

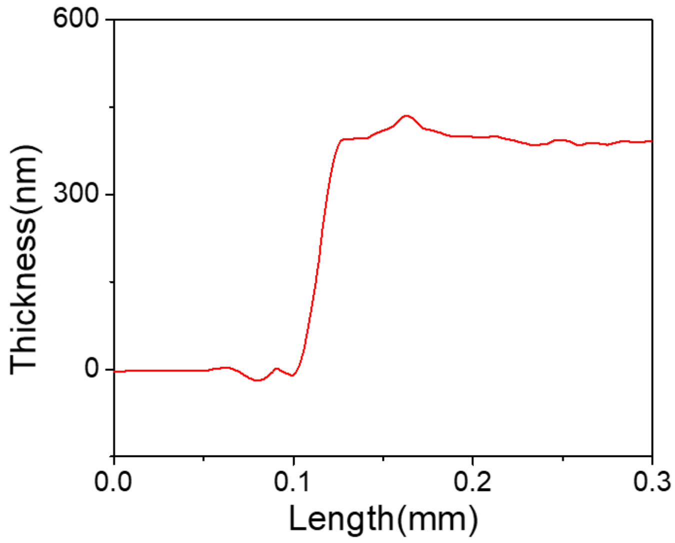

Figure S1. The thickness of sprayed graphene film tested by a probe profiler.

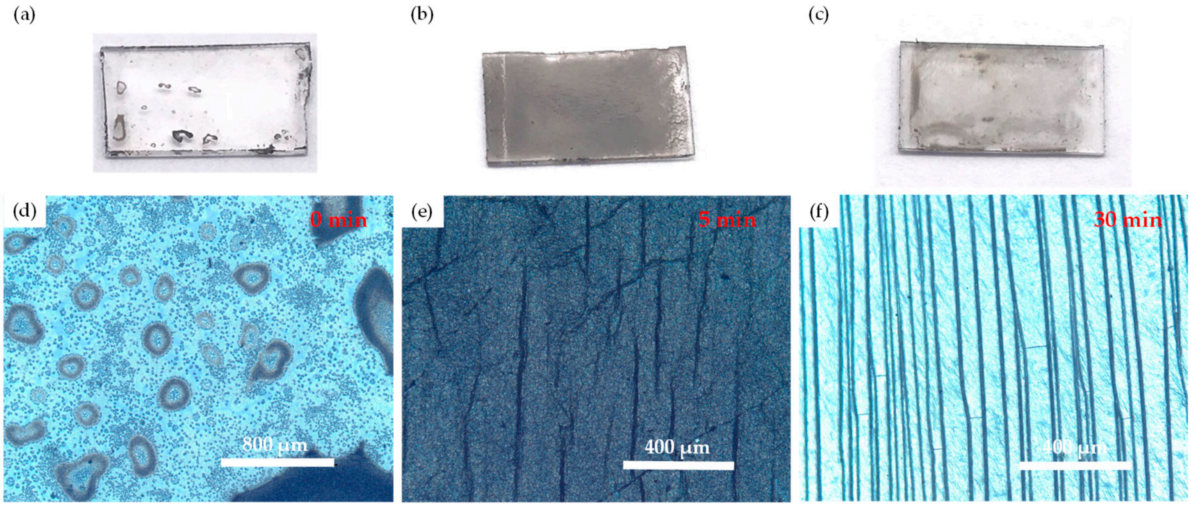

Figure S2. The surface morphology of graphene layer under different oxygen plasma treatment time. (a) - (c) Photographs of graphene strain sensors at different treatment time: 0 min, 5 min, 30 min, respectively; (d) - (f) Optical images of sensors with different oxygen plasma treatment time (0 min, 5 min, 30 min) after 10 cyclic measurement of 0-2% strain, of which the substrates are all pre-stretched under 0~ 10% strain for 10 cycles.

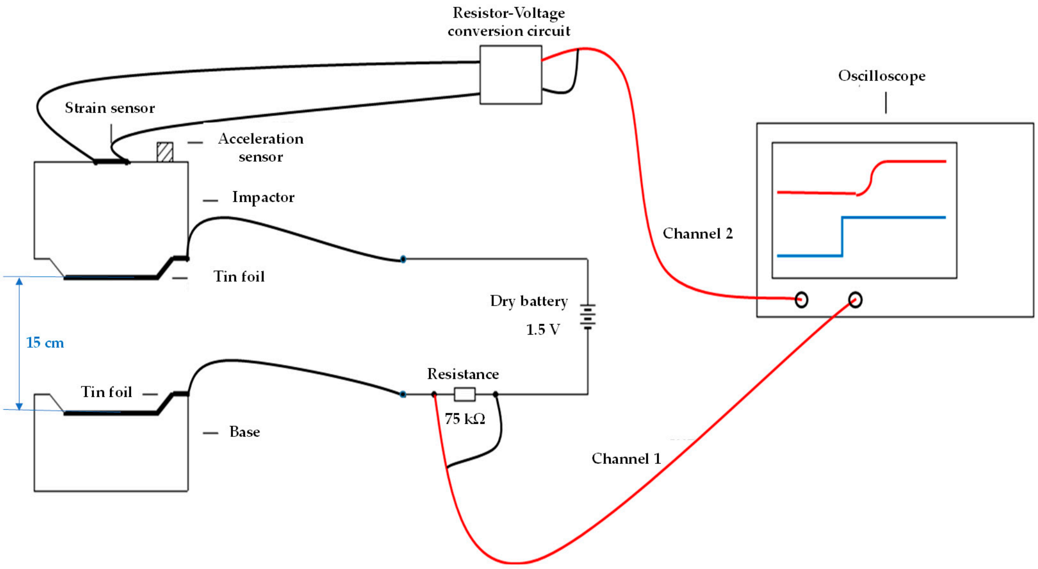

Figure S3. Schematic diagram of self-designed experimental equipment for response time test. The impactor and the base are made by the same rigid insulating material. The height of the impactor is about 5 cm and the impactor is released at the distance of 15 cm from the center of the base.

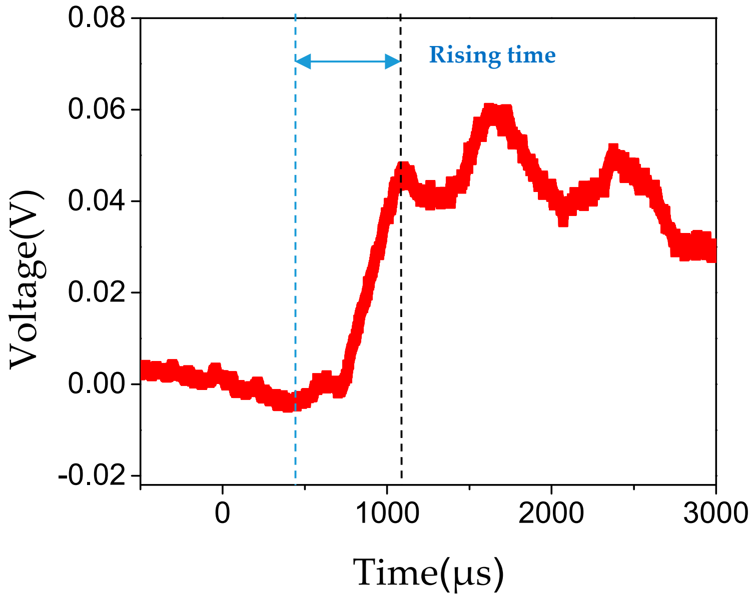

Figure S4. The whole progress of graphene strain sensor to output the response signal. The range of the rising time is about 400 to 650 μs under the impact acceleration of 500 g.

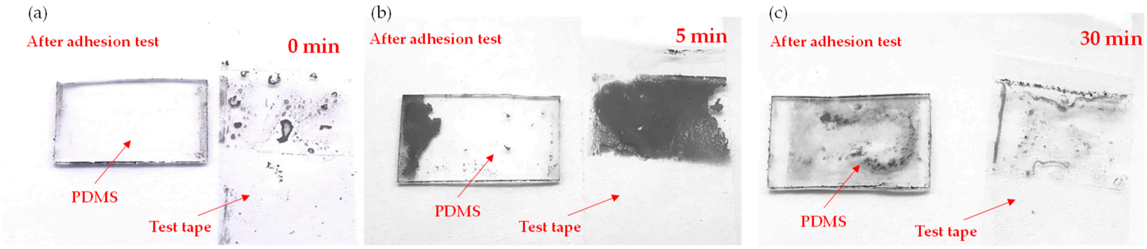

Figure S5. Interfacial bonding strength test under different oxygen plasma treatment time. (a) - (c) The treatment time of the sample is: 0 minute, 5 minutes, 30 minutes, respectively. The test tape was # 3M 600 by Scotch and 0.5N force was applied evenly on the effective area of the tape for 5 seconds. The tape was tore by the stretcher (INSTRON 3380) at a uniform speed of 0.08 mm/s.
